# ARPES Signatures of Trions in van der Waals Materials

**DOI:** 10.1021/acs.nanolett.6c00729

**Published:** 2026-04-24

**Authors:** Giuseppe Meneghini, Maja Löwe, Raul Perea-Causin, Jan Philipp Bange, Wiebke Bennecke, Marcel Reutzel, Stefan Mathias, Ermin Malic

**Affiliations:** † Department of Physics, Philipps-Universität Marburg, D-35032 Marburg, Germany; ‡ mar.quest|Marburg Center for Quantum Materials and Sustainable Technologies, Hans-Meerwein-Straße 6, D-35032 Marburg, Germany; § AlbaNova University Center, Department of Physics, 7675 Stockholm University, 11421 Stockholm, Sweden; ∥ I. Physikalisches Institut, Georg-August-Universität Göttingen, 37077 Göttingen, Germany

**Keywords:** van der Waals heterostructures, ARPES signature of trions, excitons, trions

## Abstract

Angle-resolved photoemission spectroscopy (ARPES) has recently emerged as a direct probe of excitonic correlations in two-dimensional semiconductors, resolving their dispersion and dynamics in energy–momentum space, including dark exciton states inaccessible to optical techniques. However, the ARPES fingerprint of charged excitons (trions), which plays a key role in all doped and gated two-dimensional (2D) material systems, has remained unknown so far. We present a first theoretical analysis of trion signatures in monolayer transition-metal dichalcogenides, highlighting how the additional charge carrier modifies the spectral position and shape relative to neutral excitons in ARPES spectra. Interestingly, we further predict that mass-imbalanced trions yield a characteristic double-peak structure, clearly separated in energy and line shape from neutral excitons. The predicted temperature dependence of these features offers guidance for experimental investigations aimed at identifying trionic states, thereby establishing a framework for ARPES studies of many-body Coulomb complexes in doped 2D semiconductors.

## Graphical abstract

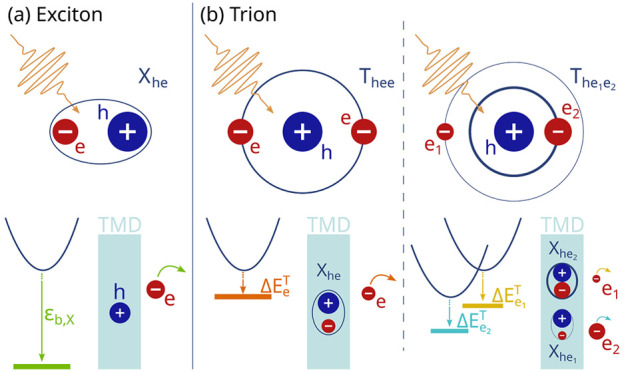

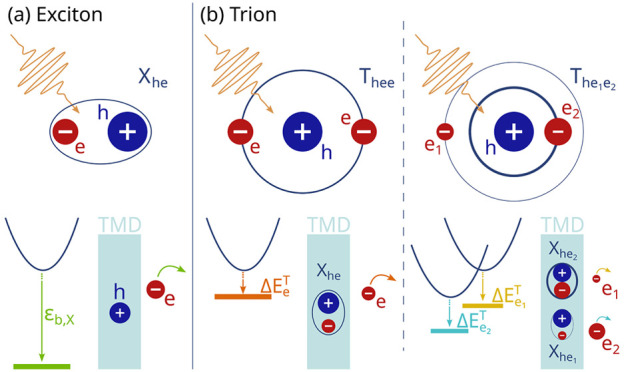

Angle-resolved photoemission spectroscopy (ARPES) has become an indispensable tool for probing the electronic structure of semiconductors.^1–4^ In two-dimensional (2D) materials, the reduced screening allows excitonic resonances to be directly observed.^5–11^ In contrast to optical techniques, ARPES provides simultaneous access to both photoelectron energy and momentum, enabling a direct detection of momentum-dark excitons^4,6,10,12–16^ that otherwise can appear only indirectly through typically weak phonon sidebands in photoluminescence.^17–21^ This ability to map the full Brillouin zone makes ARPES uniquely suited to investigate the relaxation dynamics of photoexcited quasiparticles, which govern the optical response and device performance of van der Waals heterostructures.^22^ While ARPES signatures of neutral excitons in transition-metal dichalcogenides (TMDs) have been well established, much less is known about doped systems, where at low temperature and carrier density the response is dominated by charged excitons (trions).^23–31^

In this work, we address this knowledge gap through a microscopic and material-specific analysis of photoemission spectra of electrons bound to excitons in doped TMD monolayers. Taking WSe_2_ as a representative case and focusing on n-type doping, we reveal the spectral position and shape of trion signatures in ARPES. We predict photoemission resonances arising from trions to be located one electron–exciton binding energy (tens of millielectronvolts) below the conduction band minimum (Figure 1b). Thus, the trion signal is clearly separated from the excitonic ARPES resonance, whose spectral position is lowered by the exciton binding energy (hundreds of millielectronvolts; Figure 1a). Furthermore, we show that the shape of the energy-momentum-resolved trion signal is also markedly distinct from the excitonic shape that reflects the negative curvature of the valence band. Most interestingly, we predict a characteristic double-peak feature arising from mass-imbalanced trions containing two electrons from different valleys (Figure 1b, right panel) and analyze the temperature dependence. To the best of our knowledge, this work is the first to reveal distinguishable trion features in ARPES. Our results and methodology establish a general framework for studying the photoemission fingerprints of many-body Coulomb complexes in doped 2D semiconductors.

**Figure 1:**
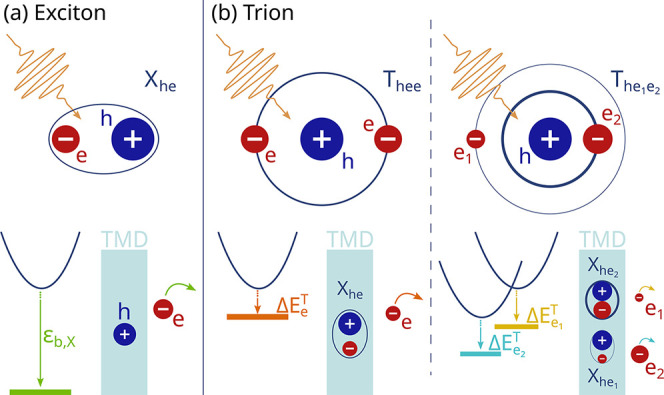
Schematic illustration of exciton and trion contributions to the ARPES spectrum of an n-doped semiconductor. Trions and excitons are denoted by $T_{h,e_{1},e_{2}}$ and $X_{h,e_{1/2}}$ , respectively, with the subindices describing the constituent electrons (e_1/2_) and holes (h). (a) Exciton case: The photoemitted electron is ejected from the material, leaving behind a hole. The signal in ARPES is located one exciton binding energy (ε_b,X_) below the conduction band minimum. (b) Trion case: For mass-balanced trions (left panel), the two electrons have a similar effective mass, so ejection of either yields the same final configuration, producing a single spectral feature in ARPES located one electron–exciton binding energy ( $\Delta E_{e}^{T}$ ) below the conduction band minimum. For mass-imbalanced trions (right panel), electrons with different effective masses have unequal binding energies to the remaining exciton ( $\Delta E_{e_{1/2}}^{T}$ ), leading to a characteristic double-peak structure in ARPES.

The ARPES intensity can be described within a time-dependent perturbation theory by the Fermi’s golden rule^1,12^

1 $$I\left(k,h\nu ;t\right)\propto \underset{if}{\sum }\left|\left\langle f,k\right|H_{\mathrm{int}}\right|i\rangle {|}^{2}N_{i}\left(t\right)\;\delta \left(\Delta E_{f,i,k}\right)$$

with |i/f⟩ and *E*_i/f_ denoting the initial/final states and their corresponding energy, with *N*_i_(*t*) as the occupation of the initial state. We focus here on the static thermalized ARPES, arising from a Boltzmann occupation of the initial state. Furthermore, Δ*E*_f,i,**k**_ = *E*_f,**k**_ – *E*_i_ – *h*ν describes the energy conservation during the photoemission process with the photon energy *h*ν and with *E*_f,**k**_ as the energy of the ejected free electron with the momentum **k**. The interaction Hamiltonian $H_{\mathrm{int}}=\underset{\alpha \beta }{\sum }M_{\alpha \beta }a_{f\alpha }^{†}a_{c\beta }$ describes the excitation of an electron from the conduction band to the free state with *a* ^(†)^ denoting the electronic creation/annihilation operator. The suffix f is used for a free state and c/v for conduction/valence band states. The optical selection rules are contained in the optical matrix element $M_{\alpha \beta }$ (see the Supporting Information, SI).

In this work, we focus on trion features in ARPES spectra, considering the exemplary case of an n-doped 2D material. The same formalism applies to p-type doping; the only distinction lies in the description of the final state, which involves a hole pair left behind by the excitation process. This results in differences in the shape and position of the corresponding spectral peaks; however, these variations are not the focus of the present study.

The initial state is a trion, which is broken into its constituents by photoemission. In the first-order process considered here, one of the trion’s electrons is photoemitted, leaving behind a bound exciton. The corresponding final state thus consists of a free electron and an excitonic state. A simultaneous ejection of both electrons or ejection of one electron leaving behind an unbound electron–hole pair could, in principle, also occur. In the latter case, we expect an exciton to be formed quickly due to the strong attractive Coulomb interaction. In the former case, we expect the resulting signal to be much weaker because it requires a multiphoton process.

To gain access to the exciton/trion energy landscape, we start from the Hamilton operator in the electron–hole picture, including the attractive electron–hole Coulomb interaction in the case of excitons and both electron–electron and electron–hole interactions in the case of trions. Then, we solve the respective Wannier-like Schrödinger equations for two and three interacting charges^30–34^

2 $$H_{e,h}\psi^{\mu }\left(k\right)=\varepsilon_{b,X}^{\mu }\psi^{\mu }\left(k\right),H_{e,e,h}\phi^{\eta }\left(k,p\right)=\varepsilon_{b,T}^{\eta }\phi^{\eta }\left(k,p\right)$$

with *H*_e,h_ being the Hamiltonian for the electron–hole complex, $\varepsilon_{b,X}^{\mu }$ the exciton binding energy, and ψ^μ^(**k**) the exciton wave function with the compound index μ = *n*, ξ_h_, ξ_e_ including the principal quantum number *n* of the excitonic Rydberg-like series and the electron/hole valley indices ξ_e/h_. In analogy, $H_{e_{1},e_{2},h}$ is the Hamiltonian for the electron–electron–hole complex, $\varepsilon_{b,T}^{\eta }$ the electron–electron–hole binding energy, and ϕ^η^(**k**,**p**) the eigenfunction for the bound triplet with the index $\eta =\xi_{h}^{s_{h}},\;\xi_{e_{1}}^{s_{e_{1}}},\;\xi_{e_{2}}^{s_{e_{2}}}$ specifying the valley and spin configuration of each constituent particle. In the following, the single-particle valley index is implicitly assumed inside the e/h indices in masses and energies.

We use the eigenfunctions ψ^μ^(**k**) and ϕ^η^(**k**,**p**), obtained by solving the corresponding Wannier equations, via direct diagonalization for excitons^35^ and a variational approach for trions,^31^ to transform into the exciton and trion bases, respectively. Here, we introduce the exciton operators $X_{Q}^{\mu †}=\underset{k}{\sum }\psi^{\mu *}\left(k\right)\;a_{c,k+{\mathrm{m̃}}_{e}Q}^{†}a_{v,k-{\mathrm{m̃}}_{h}Q}$ , where **k** and **Q** denote the relative and center-of-mass momentum, respectively, with the effective mass ratio ${\mathrm{m̃}}_{e/h}=m_{e/h}/\left(m_{e}+m_{h}\right)$ . To create a trion state consisting of two electrons (e_1_ and e_2_) and a hole, we introduce the trion creation operators $T_{Q}^{\eta †}=\underset{kp}{\sum }\phi^{\eta *}\left(k,p\right)\;a_{c_{1},\alpha_{e_{1}}Q+k}^{†}a_{c_{2},\alpha_{e_{2}}Q+p}^{†}a_{v,p+k-\alpha_{h}Q}$ .^30,31^ Here, **Q** denotes the center-of-mass momentum of the trion, while **k** and **p** are the corresponding relative momenta; furthermore, $\alpha_{i}=m_{i}/\left(m_{e_{1}}+m_{e_{2}}+m_{h}\right)$ with *i* ∈ [e_1_, e_2_, h], and c_*i*_ denoting the conduction band of electron e_*i*_. The exciton and trion Hamilton operators are diagonal in their corresponding basis, i.e., $H_{X}=\underset{\mu Q}{\sum }E_{Q,X}^{\mu }X_{Q}^{\mu †}X_{Q}^{\mu }$ and $H_{T}=\underset{\eta Q}{\sum }E_{Q,T}^{\eta }T_{Q}^{\eta †}T_{Q}^{\eta }$ . Here, the total exciton energy can be written as $E_{Q,X}^{\mu }=E_{g}^{\mu }+\varepsilon_{b,X}^{\mu }+\hbar^{2}Q^{2}/\left(2M_{X}^{\mu }\right)$ , with the single-particle band gap $E_{g}^{\mu }=E_{c}-E_{v}$ for the exciton state μ, where *E*_c/v_ denote the conduction/valence band energy. In analogy, $E_{Q,T}^{\eta }=E_{c_{1}}+E_{c_{2}}-E_{v}+\varepsilon_{b,T}^{\eta }+\hbar^{2}Q^{2}/\left(2M_{T}^{\eta }\right)$ describes the total trion energy, with $\varepsilon_{b,T}^{\eta }$ as the trion binding energy, obtained from the generalized Wannier equation. In addition, we introduce the electron–exciton binding energy $\Delta E_{e_{i}}^{T}=\varepsilon_{b,T}^{\eta }-\varepsilon_{b,X_{e_{j}h}}^{\mu }$ to denote the binding of a given electron e_*i*_ to a specific exciton $X_{e_{j}h}$ within the trion state (as shown in the schematic picture, Figure 1b).^30,36^ Now, we describe exciton and trion states as X_μ_ and T_η_, respectively, with μ and η labeling their valley and spin configurations, following the previously defined order of hole and electron indices.

In this work, we focus on the low-carrier-density and low-doping regime, where band renormalizations can be neglected and the few-body trion description provides an accurate representation of the system.^37^ At higher doping, the presence of a Fermi sea in the conduction band requires a description in terms of the Fermi polaron picture, leading to the emergence of two distinct branches (attractive and repulsive polarons). In this regime, many-body interactions result in a redistribution of spectral weight as well as in doping-dependent shifts and broadening of spectral features. In addition, in the presence of strong doping or electronic correlations, deviations from the parabolic band dispersion can arise. Because the ARPES signal reflects the electronic spectral function, such modifications of the band structure are directly accessible in the ARPES spectra.

In the case of excitons, we restrict to 1s states in the lowest spin-bright configuration and omit the spin index for simplicity. Solving the Wannier-like equations for excitons and trions (eq 2), we find that the lowest excitons in WSe_2_ monolayers are the momentum-dark X_KK′_ and X_KΛ_ states,^17,38^ while the lowest trions are $T_{K^{↑}K^{↓}K^{\prime ↑}}$ , $T_{K^{↑}K^{\prime ↑}\Lambda^{↑}}$ , and $T_{K^{↑}\Lambda^{↑}\Lambda^{\prime ↓}}$ (Figure 2c,d).^31^ In the absence of exchange interactions, these states are degenerate with respect to spin–valley configurations (e.g., K and K′ valleys with opposite spin have the same energy); thus, the signal is equal for degenerate spin-valley bands (e.g., $T_{K^{↑}K^{↓}K^{\prime ↑}}$ is degenerate with $T_{K^{↑}K^{↓}K^{↓}}$ ). Electron–hole exchange is known to slightly lift this degeneracy for both excitons and trions,^27,39–43^ but this typically small splitting introduces a secondary fine structure (i.e., a splitting into two nearby components), which is difficult to resolve. Thus, we have neglected this contribution in our work focusing on the analysis of the fingerprint of the main trion states in ARPES spectra.

**Figure 2:**
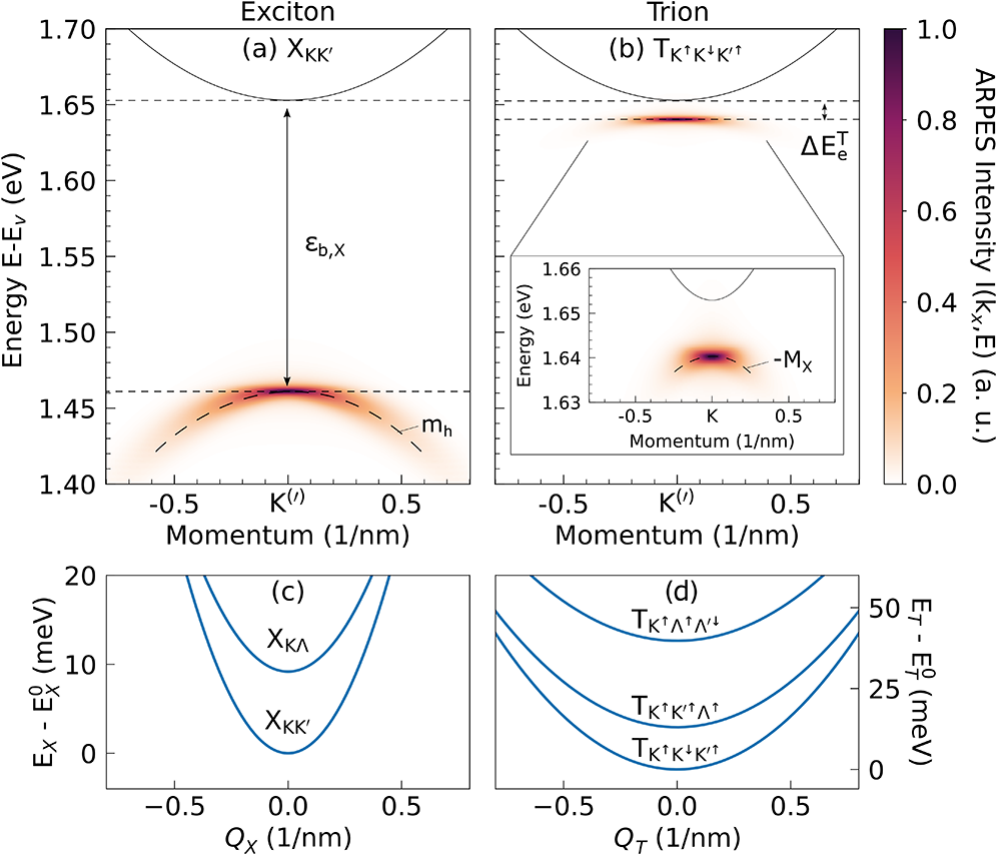
ARPES intensity *I*(*k*_*x*_,*E*) for an n-doped WSe_2_ monolayer along the *k*_*x*_ direction around the *K* ^(^′^)^ valley at *T* = 10 K, shown as a function of energy for the lowest (a) exciton X_KK′_ and (b) trion $T_{K^{↑}K^{↓}K^{\prime ↑}}$ . In both cases, the signal appears at the valley of the ejected electron, but its spectral position and shape differ. The exciton signal lies one exciton binding energy ε_b,X_ below the conduction band minimum (solid thin parabolas) and follows the valence band curvature (dashed parabola), whereas the trion signal is shifted by the much smaller electron–exciton binding energy $\Delta E_{e}^{T}$ and exhibits a nearly flat dispersion, reflecting the larger effective mass of the residual exciton. We take the valence-band maximum *E*_v_ as the reference energy level. Energy landscape of the lowest (c) excitons and (d) trions as a function of their center-of-mass momentum **Q**_X,T_, with energies measured relative to the exciton/trion ground state $E_{X/T}^{0}$ at **Q** = 0.

The initial (trion) and final (free electron + exciton) states for the ARPES process are written as |*T*_η_⟩ and |**k**⟩⊗|X_μ_⟩, with **k** being the wavevector of the emitted electron. Inserting these states in eq 1, we obtain the final equation for the ARPES signal of trions

3 $$I\left(k,h\nu \right)\propto \underset{\eta \mu Q}{\sum }|G_{Qk}^{\eta \mu }{|}^{2}N_{Q}^{\eta }\delta \left(\Delta E_{Qk}^{\eta \mu }\right)$$

where $\Delta E_{Qk}^{\eta \mu }=E_{k,e}+E_{Q-k,X}^{\mu }-E_{Q,T}^{\eta }-h\nu$ with the free electron energy *E*_**k**,e_ and the definition of exciton and trion energies given above. The photoemission signal depends directly on the trion occupation $N_{Q}^{\eta }$ in the state η and the trion center-of-mass momentum **Q** and the matrix element $|G_{Qk}^{\eta \mu }{|}^{2}$ . The latter weights are the contributions of different photoemission channels involving different electrons within the trion. These weights are determined by the internal structure of the trion wave function after tracing out the residual exciton degrees of freedom (see the SI for more details).

The derived theoretical approach is applicable to a broad class of excitonic and trionic materials. Here, we focus on the exemplary case of n-doped WSe_2_ monolayers at *T* = 10 K and solve eq 3 to obtain the ARPES intensity *I*(**k**,*E*) as a function of energy and momentum. At the cryogenic temperature, we focus only on the signature of the lowest momentum–dark exciton X_KK′_ and trion $T_{K^{↑}K^{↓}K^{\prime ↑}}$ . Figure 2 shows the ARPES intensity *I*(*k*_*x*_,*E*) along a momentum cut in the *k*_*x*_ direction. Panel a illustrates the exciton signal, which reproduces well-known photoemission features.^1,2,5,6,10,12,44–46^ The signal appears one exciton binding energy $\varepsilon_{b,X}^{\mu }$ below the conduction band minimum. At low temperatures, when the exciton distribution is sharply peaked around the center-of-mass momentum **Q** = 0, the spectral shape follows the negative valence band dispersion, in agreement with previous theoretical and experimental studies.^5,6^ Panel b presents the trion ARPES signal. Compared to neutral excitons, the trion signal is located much closer to the conduction band minimum and shows a flatter momentum dispersion, deviating from the valence band dispersion (see the inset).

Notably, while in photoluminescence trions appear one electron–exciton binding energy $\Delta E_{e_{i}}^{T}$ below the exciton peak, in ARPES the trion signal lies instead higher in energy, as it is located one $\Delta E_{e_{i}}^{T}$ (where the index e_*i*_ refers to the ejected electron) below the conduction band minimum. This difference reflects the fact that ARPES measures the energy required to remove a single electron from a bound complex. For an exciton, this corresponds to the exciton binding energy $\varepsilon_{b,X}^{\mu }$ , whereas for a trion, only the binding energy $\Delta E_{e_{i}}^{T}$ of the ejected electron to the remaining exciton matters. This follows directly from the energy and momentum conservation in eq 3. At low temperatures, where $N_{Q}^{\eta }≃\delta_{Q,0}$ , the delta function in eq 3 gives (using the previous definitions of $E_{Q,X}^{\mu }$ and $E_{Q,T}^{\eta }$ )­

4 $$E_{k,e}-h\nu =E_{c_{i}}-\left|\Delta E_{e_{i}}^{T}\right|-\frac{\hbar^{2}k^{2}}{2M_{X}}$$

demonstrating that the trion signature appears one electron–exciton binding energy $\Delta E_{e_{i}}^{T}$ below the conduction band minimum $E_{c_{i}}$ of the ejected electron e_*i*_. The shape of the photoemission signal is governed by the relatively large effective exciton mass *M*_X_, which explains the flatter momentum dependence of the trion signal. The apparent slightly negative curvature (see the inset in Figure 2b) arises from energy conservation in the photoemission process. In a realistic system, exciton and trion populations coexist with their relative occupations determined, e.g., by a Saha-type equation. However, due to their well-separated energy positions, excitonic and trionic features can be clearly distinguished.

So far, we have considered trion ARPES signals for n-doped WSe_2_ monolayers under the assumption that only one trion state is occupied. In reality, the trion landscape in this material is more complex: several low-lying states contribute, and thermal occupation at finite temperatures can considerably modify the ARPES spectra. In particular, a part of the low-energy response arises from mass-imbalanced trions composed of electrons from different valleys in the Brillouin zone exhibiting sizably distinct effective masses.^31^ Because the internal structure of these mass-imbalanced trions is more complex, the corresponding photoemission process is expected to depend sensitively on the specific trion configuration. This leads to qualitatively distinct ARPES signatures, schematically illustrated in Figure 1b. To account for this scenario, we consider a Boltzmann distribution at room temperature over the three energetically lowest trions and analyze their combined photoemission signatures.

Figure 3 displays the valley-resolved intensity *I*(*k*_*x*_,*E*) around the K′ and Λ valleys as well as the momentum-integrated ARPES intensity *I*(*E*). For the mass-balanced trions $T_{K^{↑}K^{↓}K^{\prime ↑}}$ and $T_{K^{↑}\Lambda^{↑}\Lambda^{\prime ↓}}$ , we find a single ARPES peak, respectively. The reason is that the two electrons within the trion are symmetry-equivalent: ejecting either one leaves the same residual exciton (left panel in Figure 1b). The corresponding photoemission signals appear one electron–exciton binding energy below the conduction band minimum at the momentum of the ejected electron because $\Delta E_{e_{1}}^{T}=\Delta E_{e_{2}}^{T}\equiv \Delta E_{e}^{T}$ . Quantitatively, the calculated electron–exciton binding energies are $\Delta E_{e}^{T}=12meV$ for $T_{K^{↑}K^{↓}K^{\prime ↑}}$ and $\Delta E_{e}^{T}=14meV$ for $T_{K^{↑}\Lambda^{↑}\Lambda^{\prime ↓}}$ . The situation is richer for the mass-imbalanced trion $T_{K^{↑}K^{\prime ↑}\Lambda^{↑}}$ . Because the electrons at the K′ and Λ valleys have different effective masses (0.4*m*_0_ and 0.6*m*_0_, respectively, with *m*_0_ as the electron mass^47^), their binding to the residual exciton differs considerably. As a result, ejecting a K′ electron or a Λ electron leaves behind distinct excitonic configurations with different energies (right panel in Figure 1b). This gives rise to characteristic double-peak ARPES features: we predict trion signatures to appear at $\Delta E_{K}^{T}=8meV$ below the conduction band minimum at the K′ valley and $\Delta E_{\Lambda }^{T}=31meV$ below the conduction band minimum at the Λ valley (Figure 3). We highlight the two peaks originating from the same trion in Figure 3 by enclosing the corresponding ejected electron label in an orange box.

**Figure 3:**
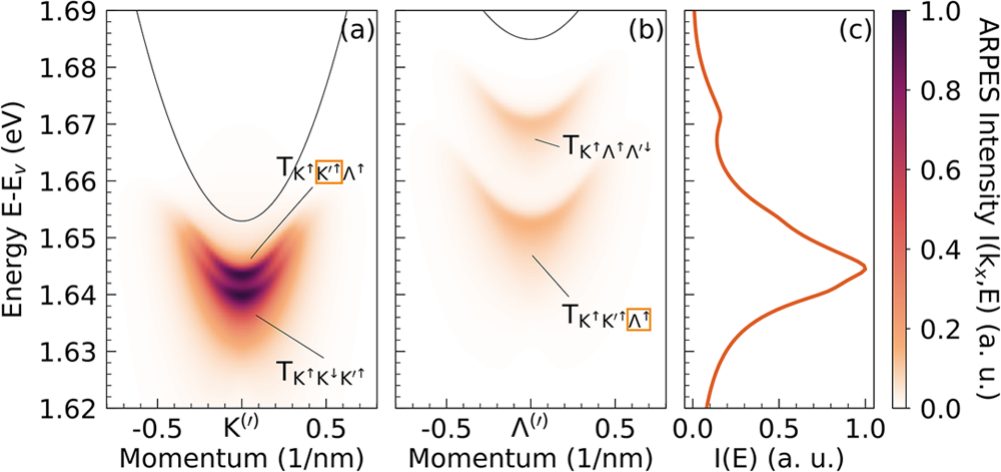
Room temperature ARPES for n-doped WSe_2_ monolayers considering a thermalized Boltzmann distribution of the three lowest trions, $T_{K^{↑}K^{↓}K^{\prime ↑}}$ , $T_{K^{↑}K^{\prime ↑}\Lambda^{↑}}$ , and $T_{K^{↑}\Lambda^{↑}\Lambda^{\prime ↓}}$ . (a and b) ARPES spectra around the K^(^′^)^ and Λ^(^′^)^ valleys, respectively, relative to the conduction band minimum (solid black line). For the mass-imbalanced trion $T_{K^{↑}K^{\prime ↑}\Lambda^{↑}}$ , the origin of the double signal is indicated by orange boxes around the corresponding ejected electron. (c) Momentum-integrated ARPES illustrating the emergence of multiple trionic resonances and the pronounced spectral broadening compared to the low-temperature case (Figure 2).

The relative intensity of the two peaks is determined by the matrix element $|G_{Qk}^{\eta \mu }{|}^{2}$ in eq 3, which contains the conditional probability of ejecting a given electron, obtained by tracing out the excitonic degrees of freedom from the trion wave function (see the SI for more details). Because the excitonic components X_KK′_ and X_KΛ_ possess different wave functions, $|G_{Qk}^{\eta \mu }{|}^{2}$ differs for each excitonic configuration, resulting in distinct emission intensities for the two channels. The momentum-integrated ARPES signal *I*(*E*), shown in Figure 3c, reveals how the combined contributions of these resonances produce a richer and more intricate peak structure compared to the excitonic case. Furthermore, by comparing Figures 2a,b and 3, we find that increasing the temperature from 10 K to room temperature markedly alters the trion signal: the features broaden both in energy and momentum, and the apparent curvature becomes positive due to the combined contributions of excitonic and trionic dispersions, as reflected in the delta function of eq 3. This behavior arises from the much broader center-of-mass momentum distribution of a thermal trion occupation.

We now examine the momentum-integrated temperature dependence of ARPES for n-doped WSe_2_ monolayers and compare them with the excitonic response. Figure 4a shows the undoped case, where the exciton signal at low temperatures is broad and dominated by a single peak. A long asymmetric tail extends to lower energies, reminiscent of the “recoil effect” in optical spectra.^48^ Such tails are intrinsic and arise from the joint constraints of momentum and energy conservation (delta function in eq 3). As the temperature increases, the spectrum broadens asymmetrically toward higher energies and a second peak emerges. These two features originate from the two lowest excitonic resonances of WSe_2_, namely, X_KK′_ and X_KΛ_ (Figure 2c). Because the two excitonic states are separated by approximately 10 meV, a sufficiently high temperature (around 100 K) is required to thermally populate the higher X_KΛ_ state and make its contribution visible in ARPES.

**Figure 4:**
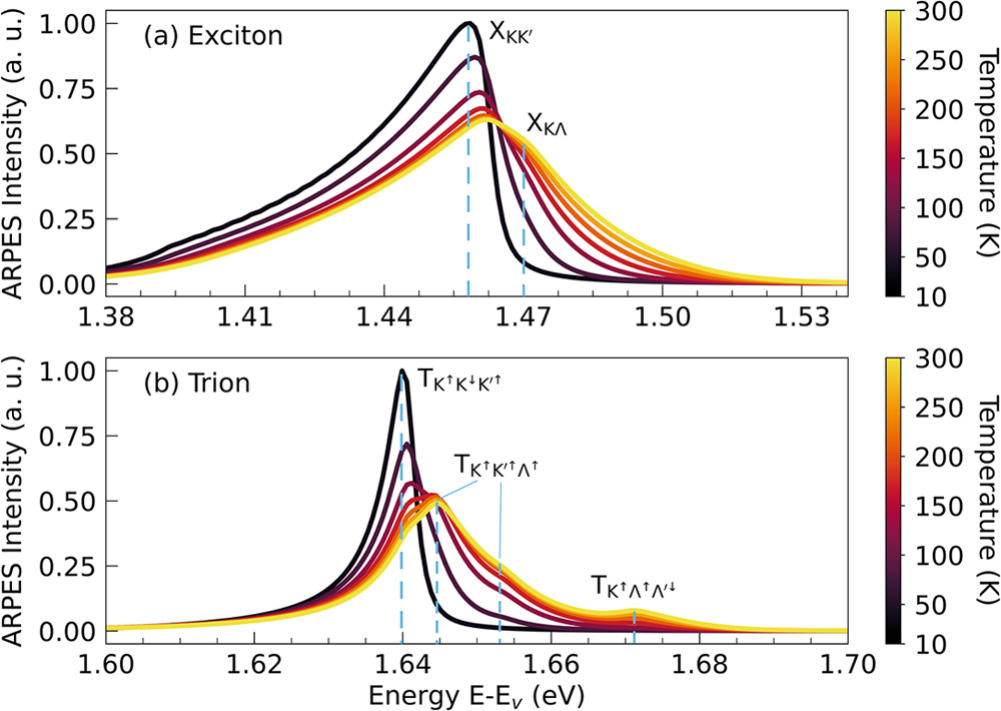
Temperature dependence of the K and Λ valley momentum-integrated ARPES for (a) excitons and (b) trions illustrating the thermal activation of higher-lying states.

Figure 4b illustrates the trion case in n-doped WSe_2_ monolayers, which exhibits a richer temperature-dependent evolution. At low temperatures, the spectrum is dominated by the energetically lowest mass-balanced trion $T_{K^{↑}K^{↓}K^{\prime ↑}}$ , resulting in a single peak. As the temperature increases, the thermal population of higher-energy trions produces a multiplet structure: four distinct subpeaks appear within a 50 meV window. They arise from the three energetically lowest trions, $T_{K^{↑}K^{↓}K^{\prime ↑}}$ , $T_{K^{↑}K^{\prime ↑}\Lambda^{↑}}$ , and $T_{K^{↑}\Lambda^{↑}\Lambda^{\prime ↓}}$ , lying close in energy (Figure 2d). Here, the mass-imbalanced trion $T_{K^{↑}K^{\prime ↑}\Lambda^{↑}}$ contributes with a double-peak, as discussed in Figure 3. Similar to the exciton case, a thermal activation is required to bridge the energy gaps between the trion states. At low temperatures, only the lowest trion contributes significantly. Around 50 K, the second trion $T_{K^{↑}K^{\prime ↑}\Lambda^{↑}}$ (≈13 meV above the lowest state) begins to contribute. Only at higher temperatures (≈100–150 K) does the third trion $T_{K^{↑}\Lambda^{↑}\Lambda^{\prime ↓}}$ (≈40 meV above the lowest state) also become important and result in an additional peak. Given that the energy separation between these features is on the order of tens of millielectronvolts, resolving them could be challenging with the energy resolution currently achievable in femtoseconds ARPES experiments.^10,11,13^ However, the characteristic double-peak structure arising from the mass-imbalanced trion appears at separate valleys and is thus accessible in experiments.

We have developed a material-specific and predictive microscopic framework for describing excitonic and trionic signatures in ARPES spectra of doped atomically thin semiconductors. By explicitly computing the momentum-resolved response of the exemplary n-doped WSe_2_ monolayer, we answer the still unresolved question of how charged excitons manifest in ARPES. We demonstrate that trions generate robust fingerprints, including specific shifts in energy and a modified spectral shape with respect to excitonic signatures. In particular, we predict a distinctive double-peak feature for mass-imbalanced trions and a thermally activated multiplet of resonances, providing quantitative criteria for their unambiguous detection in ARPES experiments. The developed approach establishes a general framework for studying many-body Coulomb complexes in doped 2D semiconductors using ARPES.

## Supplementary Material



## Notes

Derivation of the main Hamiltonians in the exciton and trion formalism, derivation of the ARPES intensity equation, and details on the quantities introduced in the main text (PDF)
