# Supplementary material for: ARPES Signatures of Trions in van der Waals Materials
Source: Nano Lett. 2026 Apr 24;26(17):5866–72. doi: 10.1021/acs.nanolett.6c00729 (PMC13154362; doi:10.1021/acs.nanolett.6c00729)
Supplement: Supplementary file 1 [file nl6c00729_si_001.pdf]

ARPES signatures of trions in van der Waals materials
Supplementary Information

Giuseppe Meneghini,[1, 2, *] Maja Löwe,[1, 2] Raul Perea-Causin,[3] Jan Philipp Bange,[4] Wiebke Bennecke,[4] Marcel Reutzel,[1, 2] Stefan Mathias,[4] and Ermin Malic[1, 2]

[1] *Department of Physics, Philipps-Universität Marburg, D-35032 Marburg, Germany*
[2] *mar.quest — Marburg Center for Quantum Materials and Sustainable Technologies, Hans-Meerwein-Straße 6, D-35032 Marburg, Germany*
[3] *Department of Physics, Stockholm University, AlbaNova University Center, Stockholm, Sweden*
[4] *I. Physikalisches Institut, Georg-August-Universität Göttingen, Göttingen, Germany*

THEORETICAL APPROACH

In order to study the ARPES signal, we first calculate microscopically exciton and trion energies and wavefunctions by solving the respective Wannier-like Schrödinger equations for two and three interacting charges.

Exciton band structure

The Wannier equation for exciton reads[1,2]

$$(\varepsilon^c_{\mathbf{k}_e} - \varepsilon^v_{\mathbf{k}_h})\Psi^\mu(\mathbf{k}_e, \mathbf{k}_h) - \sum_{\mathbf{q}} V_{\mathbf{q}} \Psi^\mu(\mathbf{k}_e + \mathbf{q}, \mathbf{k}_h + \mathbf{q}) = E^\mu_{\mathbf{k}_e,\mathbf{k}_h} \Psi^\mu(\mathbf{k}_e, \mathbf{k}_h) \tag{1}$$

To exploit the translational symmetry, it is useful to rewrite the problem in terms of center-of-mass and relative momenta, $\mathbf{Q} = \mathbf{k}_e - \mathbf{k}_h$ and $\mathbf{k} = (m_e/M_X)\mathbf{k}_h + (m_h/M_X)\mathbf{k}_e$ with the total mass $M_X = m_e + m_h$. Furthermore, $\varepsilon^c_{\mathbf{k}_e} - \varepsilon^v_{\mathbf{k}_h} = \hbar^2(\mathbf{k}^2/(2m_r) + \mathbf{Q}^2/(2M_X)) + E_{gap}$ with $E_{gap} = E_c - E_v$ corresponds to the energy difference between the conduction and the valence bands. Now, the problem can be decoupled into a center-of-mass and a relative momentum wavefunction $\Psi^\mu(\mathbf{k}_e, \mathbf{k}_h) = \psi^\mu(\mathbf{k})\eta(\mathbf{Q})$. By solving the eigenvalue problem of the relative momentum part

$$\frac{\hbar^2\mathbf{k}^2}{2m^r}\psi^\mu(\mathbf{k}) - \sum_{\mathbf{q}} V_{\mathbf{q}}\psi^\mu(\mathbf{k}+\mathbf{q}) = \varepsilon^\mu_{b,X}\psi^\mu(\mathbf{k}) \tag{2}$$

we obtain exciton binding energies $\varepsilon^\mu_{b,X}$ and relative momentum wave functions $\psi^\mu(\mathbf{k})$ with the exciton index μ. Here, $m^r = (1/m_c + 1/m_v)^{-1}$ is the reduced mass with $m_{c/v}$ being the effective mass of the conduction/valence band. Here, we used the effective Coulomb potential with a static dielectric screening

$$V_{\mathbf{q}} = \frac{e_0^2}{2\epsilon_0 A q \epsilon_s(q)} \quad \text{with } \epsilon_s(q) = \kappa\frac{1-\delta e^{-\alpha d q}}{1+\delta e^{-\alpha d q}} \tag{3}$$

where $\kappa = \sqrt{\epsilon_{TMD,\parallel}\epsilon_{TMD,\perp}}$, $\delta = (\kappa - \epsilon_{bg})/(\kappa + \epsilon_{bg})$, and $\alpha = \sqrt{\epsilon_{TMD,\parallel}/\epsilon_{TMD,\perp}}$ containing the dielectric constants of the background ϵ_{bg} and of the TMD layer ϵ_{TMD}. The total energy of the exciton reads $E^\mu_{\mathbf{Q}} = E_{gap} + \varepsilon^\mu_{\mathbf{b},\mathbf{X}} + \hbar^2\mathbf{Q}^2/(2M_X)$. With the introduction of exciton operators

$$X^{\mu\dagger}_{\mathbf{Q}} = \sum_{\mathbf{k}} \psi^{\mu *}(\mathbf{k}) a^\dagger_{c,\mathbf{k}+(m_e/M_X)\mathbf{Q}} a_{v,\mathbf{k}-(m_h/M_X)\mathbf{Q}}, \tag{4}$$

expressed in terms of electron annihilation (creation) operators for conduction/valence band $a^{(\dagger)}_{c/v}$, we obtain the excitonic Hamiltonian $H_X = \sum_{\mu,\mathbf{Q}} E^\mu_{\mathbf{Q}} X^{\mu\dagger}_{\mathbf{Q}} X^\mu_{\mathbf{Q}}$.

Trion band structure

We introduce the generalized Wannier equation for a system of three particles (e_1, e_2, h)—two electrons and one hole, relevant for n-doped materials. By separating the problem into center-of-mass and relative coordinates, as done for excitons,

$$\begin{aligned} \mathbf{Q} &= \mathbf{k}_{e_1} + \mathbf{k}_{e_2} - \mathbf{k}_h \\ \mathbf{k} &= (m_{e_2}/M_T + m_h/M_T)\mathbf{k}_{e_1} - m_{e_1}/M_T(\mathbf{k}_{e_2} - \mathbf{k}_h) \\ \mathbf{k}' &= (m_{e_1}/M_T + m_h/M_T)\mathbf{k}_{e_2} - m_{e_2}/M_T(\mathbf{k}_{e_1} - \mathbf{k}_h) \end{aligned} \tag{5}$$

we obtain the following eigenvalue problem[3]

$$\frac{\hbar^2\mathbf{k}^2}{2m^r_{e_1,h}} + \frac{\hbar^2\mathbf{k}'^2}{2m^r_{e_2,h}} + \frac{\hbar^2\mathbf{k}\cdot\mathbf{k}'}{m^r_h}\phi^\eta(\mathbf{k},\mathbf{k}') + \sum_{\mathbf{q}} V_{\mathbf{q}}(\phi^\eta(\mathbf{k}+\mathbf{q},\mathbf{k}'-\mathbf{q}) - \phi^\eta(\mathbf{k}+\mathbf{q},\mathbf{k}') - \phi^\eta(\mathbf{k},\mathbf{k}'-\mathbf{q})) = \varepsilon^\eta_{b,T}\phi^\eta(\mathbf{k},\mathbf{k}') \tag{6}$$

with $m^r_{i,j} = (1/m_i + 1/m_j)^{-1}$ as the reduced mass of the pair i, j, furthermore, with $\phi^\eta(\mathbf{k},\mathbf{k}')$ and $\varepsilon^\eta_{b,T}$ denoting the binding energy and the wavefunction for the trion state η, respectively.

We assume the following real-space variational ansatz for the relative wavefunction[3]

$$\phi^\eta(\mathbf{r},\mathbf{r}') = \mathcal{N}\left(e^{-|\mathbf{r}|/a}e^{-|\mathbf{r}'|/b} + Ce^{-|\mathbf{r}|/c}e^{-|\mathbf{r}'|/d}\right) \tag{7}$$

to minimize the total trion energy with variational parameters a, b, c, d, C. Note that this ansatz is an asymmetric analog[3] of the symmetrized product of hydrogenic functions[4] which allows for different electron orbitals, e.g. due to mass imbalance. The total trion energy with respect to the unbound triplet as function of the trion center-of-mass momentum reads $E^\eta_{\mathbf{Q}} = E_{c_1} + E_{c_2} - E_v + \varepsilon^\eta_{b,T} + \hbar^2\mathbf{Q}^2/(2M_T)$, where E_{c_i} is the conduction band energy for the electron e_i and E_v is the valence band energy for the hole. After a basis transformation by introducing the trion operator

$$T^{\eta\dagger}_{\mathbf{Q}} = \sum_{\mathbf{k},\mathbf{p}} \phi^{\eta*}(\mathbf{k},\mathbf{p}) a^\dagger_{c1,(m_{e_1}/M_T)\mathbf{Q}+\mathbf{k}} a^\dagger_{c2,(m_{e_2}/M_T)\mathbf{Q}+\mathbf{p}} a_{v,\mathbf{k}+\mathbf{p}-(m_h/M_T)\mathbf{Q}}, \tag{8}$$

we obtain a trion Hamiltonian $H = \sum_{\mathbf{Q},\eta} \varepsilon^\eta_{\mathbf{Q}} T^{\eta\dagger}_{\mathbf{Q}} T^\eta_{\mathbf{Q}}$.

Trion ARPES signal

We outline here the derivation of the ARPES signal generated by trions. For convenience, we collect exciton and trion quantum numbers into superscripts $\mu = (n, \xi_e, \xi_h)$ and $\eta = (n, \xi_{e_1}, \xi_{e_2}, \xi_h)$, where the valley index ξ includes both momentum and spin. In this notation, the corresponding effective masses depend on the specific valley configuration, i.e., $M_X = M^\mu_X$ and $M_T = M^\eta_T$. The calculation is based on the three-step model[5], where the leading contribution to the ARPES signal follows from Fermi's golden rule:

$$\mathcal{I}(\mathbf{p}, h\nu) \propto \sum_{i,f} |\langle f, \mathbf{k}| H_{int} |i\rangle|^2 N_i \delta(E_{f\mathbf{p}} - E_i - h\nu)\,, \tag{9}$$

where $|i\rangle$ and $|f\rangle$ denote the initial and final many-body states of the system. In the following, $\mathbf{p}$ describes the momentum component parallel to the sample surface, which is conserved during photoemission. We consider initial states given by trion eigenstates $\left|T^\eta_{\mathbf{Q}}\right\rangle$ with the dispersion $E^\eta_{\mathbf{Q}}$, and final states consisting of an emitted free electron and a residual exciton, $|\mathbf{p}\rangle \otimes |X^\mu_{\mathbf{P}}\rangle$, where $|\mathbf{p}\rangle$ is a plane wave. With these definitions, Eq. (9) becomes

$$\mathcal{I}(\mathbf{p}, h\nu) \propto \sum_{\eta,\mu,\mathbf{Q},\mathbf{P}} \left|\langle X^\mu_{\mathbf{P}}, \mathbf{p}| H_{int} \left|T^\eta_{\mathbf{Q}}\right\rangle\right|^2 N^\eta_{\mathbf{Q}} \delta\Big(E_{\mathbf{p},e} + E^\mu_{\mathbf{P},X} - E^\eta_{\mathbf{Q},T} - h\nu\Big)\,, \tag{10}$$

where $\mathbf{Q}$ and $\mathbf{P}$ are the trion and exciton center-of-mass momenta, respectively, and $E_{\mathbf{p},e}$ is the free-electron kinetic energy.

We model the photoemission process using the electron–light coupling

$$H_{int} = \sum_{\mathbf{p},\mathbf{k}} \mathcal{M}_{\mathbf{pk}}\, a^{\dagger}_{f,\mathbf{p}} a^{c,\mathbf{k}}, \tag{11}$$

where the optical matrix element is approximated as $\mathcal{M}_{\mathbf{pk}} = -i\hbar \langle \mathbf{p}| \nabla |\mathbf{k}\rangle \approx \delta_{\mathbf{k}_{\parallel},\mathbf{p}} M$, assuming a band and momentum-independent dipole matrix element M within the conduction band, and $a^{(\dagger)}_{\lambda,\mathbf{k}}$ annihilation(creation) operator for destroying (creating) and electron in the state λ (with f free, c conduction band) and momentum $\mathbf{k}$. Evaluating $\langle X^{\mu}_{\mathbf{P}}, \mathbf{p}|\, H_{int} \left| T^{\eta}_{\mathbf{Q}} \right\rangle$ by substituting the explicit exciton and trion operators in terms of electronic operators (Eqs (4),(8)), yields

$$\sum_{\mathbf{q},\mathbf{q}',\mathbf{k},\mathbf{k}'} \psi^{\mu *}(\mathbf{q})\, M\, \phi^{\eta}(\mathbf{k},\mathbf{k}') \left\langle \hat{A}^{\mu\eta}_{\mathbf{pk'qq'QP}} \right\rangle,$$

with $\hat{A}^{\mu\eta}_{\mathbf{pk'qq'QP}} = a_{f,\mathbf{p}} a^{\dagger}_{v,\mathbf{q}-(m^{\mu}_{h}/M^{\mu}_{X})\mathbf{P}} a_{c,\mathbf{q}+(m^{\mu}_{e}/M^{\mu}_{X})\mathbf{P}} a^{\dagger}_{f,\mathbf{q}'} a_{c,\mathbf{q}'} a^{\dagger}_{c_1,(m^{\eta}_{e_1}/M^{\eta}_{T})\mathbf{Q}+\mathbf{k}} a^{\dagger}_{c_2,(m^{\eta}_{e_2}/M^{\eta}_{T})\mathbf{Q}+\mathbf{k}'} a_{v,\mathbf{k}+\mathbf{k}'-(m^{\eta}_{h}/M^{\eta}_{T})\mathbf{Q}}$.

The expectation value is non-zero only for operator in which each creation operator is paired with a corresponding annihilation operator acting on the same state. For fermions, any attempt to create (or annihilate) two particles in the same state yields zero by the Pauli exclusion principle. As a result, only these paired combinations contribute, producing delta functions that fix the relevant momenta and quantum numbers. This leads to

$$\left\langle \hat{A}^{\mu\eta}_{\mathbf{pk'qq'QP}} \right\rangle = \mathcal{G}^{\mu\eta}_{1}(\mathbf{P},\mathbf{Q},\mathbf{p}) - \mathcal{G}^{\mu\eta}_{2}(\mathbf{P},\mathbf{Q},\mathbf{p}),$$

with

$$\mathcal{G}^{\mu\eta}_{1}(\mathbf{P},\mathbf{Q},\mathbf{p}) = \delta_{\mathbf{p},\mathbf{q}'} \delta_{\mathbf{q}-(m^{\mu}_{h}/M^{\mu}_{X})\mathbf{P},\, \mathbf{k}+\mathbf{k}'-(m^{\eta}_{h}/M^{\eta}_{T})\mathbf{Q}} \delta_{\mathbf{q}+(m^{\mu}_{h}/M^{\mu}_{X})\mathbf{P},\, (m^{\eta}_{e_2}/M^{\eta}_{T})\mathbf{Q}+\mathbf{k}'} \delta_{\mathbf{q}',\, (m^{\eta}_{e_1}/M^{\eta}_{T})\mathbf{Q}+\mathbf{k}} \delta_{c^{\mu},c^{\eta}_{2}},$$
$$\mathcal{G}^{\mu\eta}_{2}(\mathbf{P},\mathbf{Q},\mathbf{p}) = \delta_{\mathbf{p},\mathbf{q}'} \delta_{\mathbf{q}-(m^{\mu}_{h}/M^{\mu}_{X})\mathbf{P},\, \mathbf{k}+\mathbf{k}'-(m^{\eta}_{h}/M^{\eta}_{T})\mathbf{Q}} \delta_{\mathbf{q}+(m^{\mu}_{h}/M^{\mu}_{X})\mathbf{P},\, (m^{\eta}_{e_1}/M^{\eta}_{T})\mathbf{Q}+\mathbf{k}} \delta_{\mathbf{q}',\, (m^{\eta}_{e_2}/M^{\eta}_{T})\mathbf{Q}+\mathbf{k}'} \delta_{c^{\mu},c^{\eta}_{1}}.$$

These terms correspond to ejecting the electron e_1 or e_2, respectively, leaving behind the remaining electron–hole pair as an exciton. Performing the momentum summations and inserting the result into Eq. (10) gives the final result

$$\mathcal{I}(\mathbf{p}, h\nu) \propto \sum_{\eta,\mu,\mathbf{Q}} \left| \mathcal{G}^{\eta\mu}_{\mathbf{Qp}} \right|^{2} N^{\eta}_{\mathbf{Q}} \delta\Big(E_{\mathbf{p},e} + E^{\mu}_{\mathbf{Q}-\mathbf{p},X} - E^{\eta}_{\mathbf{Q},T} - h\nu\Big), \tag{12}$$

where $\mathcal{G}^{\eta\mu}_{\mathbf{Qp}} = G^{\mu\eta}_{1}(\mathbf{Q},\mathbf{p}) - G^{\mu\eta}_{2}(\mathbf{Q},\mathbf{p})$, with

$$G^{\mu\eta}_{1}(\mathbf{Q},\mathbf{p}) = \sum_{\mathbf{k}} M\, \psi^{\mu *}\left(\mathbf{k} + \frac{m^{\mu}_{e_2}}{M^{\mu}_{X}}\mathbf{p} - \left(\frac{m^{\eta}_{e_1} + m^{\eta}_{h}}{M^{\eta}_{T}} - \frac{m^{\mu}_{h}}{M^{\mu}_{X}}\right)\mathbf{Q}\right) \phi^{\eta}\left(\mathbf{p} - \frac{m^{\eta}_{e_1}}{M^{\eta}_{T}}\mathbf{Q}, \mathbf{k}\right) \delta_{e_{\mu},e_2},$$

$$G^{\mu\eta}_{2}(\mathbf{Q},\mathbf{p}) = \sum_{\mathbf{k}} M\, \psi^{\mu *}\left(\mathbf{k} + \frac{m^{\mu}_{e_1}}{M^{\mu}_{X}}\mathbf{p} - \left(\frac{m^{\eta}_{e_2} + m^{\eta}_{h}}{M^{\eta}_{T}} - \frac{m^{\mu}_{h}}{M^{\mu}_{X}}\right)\mathbf{Q}\right) \phi^{\eta}\left(\mathbf{k}, \mathbf{p} - \frac{m^{\eta}_{e_2}}{M^{\eta}_{T}}\mathbf{Q}\right) \delta_{e_{\mu},e_1}.$$

The final result shows that the probability of ejecting an electron, $\left| \mathcal{G}^{\eta\mu}_{\mathbf{Qp}} \right|^{2}$, arises from the combination of the two possible emission channels, each weighted by its corresponding $G^{\mu\eta}_{1/2}(\mathbf{Q},\mathbf{p})$. These coefficients encode the conditional probability of finding a given electron in the vicinity of the residual exciton formed by the non-ejected electron and are obtained by tracing out the excitonic degrees of freedom from the trion wavefunction. Consequently, the greater the mass imbalance within the trion, the more distinct the two coefficients $G^{\mu\eta}_{1/2}(\mathbf{Q},\mathbf{p})$ become.

* giuseppe.meneghini@physik.uni-marburg.de

[1] Gunnar Berghäuser and Ermin Malic, "Analytical approach to excitonic properties of mos 2," Physical Review B **89**, 125309 (2014).
[2] Samuel Brem, Malte Selig, Gunnar Berghaeuser, and Ermin Malic, "Exciton relaxation cascade in two-dimensional transition metal dichalcogenides," Scientific reports **8**, 1–8 (2018).
[3] Raul Perea-Causin, Samuel Brem, Ole Schmidt, and Ermin Malic, "Trion photoluminescence and trion stability in atomically thin semiconductors," Physical Review Letters **132**, 036903 (2024).
[4] Timothy C. Berkelbach, Mark S. Hybertsen, and David R. Reichman, "Theory of neutral and charged excitons in monolayer transition metal dichalcogenides," Phys. Rev. B **88**, 045318 (2013).
[5] Andrea Damascelli, Zahid Hussain, and Zhi-Xun Shen, "Angle-resolved photoemission studies of the cuprate superconductors," Reviews of modern physics **75**, 473 (2003).
